# The Impact of Frailty, Oropharyngeal Dysphagia and Malnutrition on Mortality in Older Patients Hospitalized for Covid-19

**DOI:** 10.14336/AD.2023.0425-2

**Published:** 2024-04-01

**Authors:** Alberto Martín-Martínez, Paula Viñas, Irene Carrillo, Josep Martos, Pere Clavé, Omar Ortega

**Affiliations:** ^1^Gastrointestinal Physiology Laboratory CIBERehd CSdM-UAB, Hospital de Mataró, Consorci Sanitari del Maresme, Universitat Autònoma de Barcelona, 08304 Mataró, Spain.; ^2^Centro de Investigación Biomédica en Red de Enfermedades Hepáticas y Digestivas (CIBERehd), 28029 Madrid, Spain.; ^3^Department of Geriatrics, Hospital de Mataró, Consorci Sanitari del Maresme, 08304 Mataró, Spain.

**Keywords:** Frailty, older patients, geriatrics, COVID-19, swallowing disorders, malnutrition

## Abstract

COVID-19 hospital mortality is higher among older patients through as yet little-known factors. We aimed to assess the effect of frailty (FR), oropharyngeal dysphagia (OD) and malnutrition (MN) on mortality in hospitalized COVID-19 older patients. Prospective cohort study of older patients (>70 years) with COVID-19 admitted to a general hospital from April 2020 to January 2021. Patients were evaluated on admission, discharge and at 1- and 3-months follow up. FR was assessed with FRAIL-VIG, OD with Volume-Viscosity Swallowing Test and MN with GLIM criteria. Clinical characteristics and outcomes, including intra-hospital, 1- and 3-month mortality, were analyzed. 258 patients were included (82.5±7.6 years; 58.9% women); 66.7% had FR (mild 28.7%, moderate 27.1% and severe 10.9%); 65.4%, OD and 50.6%, MN. OD prevalence increased from non-FR patients through the severity levels of FR: mild, moderate and severe (29.8%, 71.6%, 90.0%, 96.2%; p<0.0001, respectively), but not that of MN (50.6%, 47.1%, 52.5%, 56.0%). Mortality over the whole study significantly increased across FR categories (9.3% non-FR; 23.0% mild; 35.7% moderate; 75.0% severe; p<.001). Functionality (Barthel pre-admission, HR=0.983, CI-95%:0.973-0.993; p=0.001), OD (HR=2.953, CI-95%:0.970-8.989; p=0.057) and MN (HR=4.279, CI-95%:1.658-11.049; p=0.003) were independent risk factors for intra-hospital mortality. FR, OD and MN are highly prevalent conditions in older patients hospitalized with COVID-19. Functionality, OD and MN were independent risk factors for intra-hospital mortality.

## INTRODUCTION

COVID-19 disease, caused by Severe Acute Respiratory Syndrome Coronavirus (SARS-CoV-2), was declared in 2020 by the World Health Organization (WHO). It caused high mortality and severe illness, with fever, cough, sore throat, fatigue, and respiratory distress [1,2]. SARS-COV-2 infection causes a cytokine storm leading to increased energy expenditure and disruption of mechanisms affecting the immune system [3,4]. COVID-19 was especially severe in older patients, causing high mortality rates [5].

Older individuals are more susceptible to severe clinical outcomes and fatality associated with COVID-19 compared with other age groups [6]. A study on age-specific mortality in COVID-19 patients found that the infection-to-fatality ratio (COVID-19 deaths to total SARS-CoV2 infections) ranged from 0.001% (95% Credible Interval [CrI]: 0-0.001) in those aged 5-9 years to 8.29% (95%CrI: 7.11-9.59%) in those aged 80+, with an increase of 0.59% with each 5-year age increase [7]. Old age is a significant predictor of poor prognosis, suggesting that undefined aging-related mechanisms may be integral elements in the severity of this disease. Early identification on admission of clinical factors causing this poor outcome in older COVID-19 might help understand the natural history of the disease and develop proactive treatments to mitigate these negative clinical outcomes.

Frailty (FR) is defined as a multidimensional geriatric syndrome characterized by the cumulative impairment of multiple body systems or functions [8,9,10]. It is highly prevalent with increasing age and patients have increased risk of adverse health outcomes such as disability and dependency, institutionalization, falls and fractures, increased comorbidities, hospitalization and mortality [11,12]. In the COVID-19 pandemic, FR had a detrimental impact on older adults and the need for assessing FR among them has become more evident. However, there is no gold standard for FR assessment, and no specific measuring tool for COVID-19 patients. FR is associated with increased illness severity, intensive care unit admission, invasive mechanical ventilation, longer hospital stay and death in COVID-19 patients [5]. It has been recommended to pay more attention to FR screening among COVID-19 patients to aid prognosis and decision-making [5]. In turn, oropharyngeal dysphagia (OD) is closely related to aging, impaired functionality, FR, COVID-19 and impaired outcomes such as malnutrition (MN) [13,14]. A study from a general hospital concluded that prevalence of OD (51.7%) and MN (45.5%; 10.1±5.0kg weight loss) in hospitalized patients with COVID-19 was very high [13]. OD was associated with greater 6-month mortality, was an independent risk factor for MN and independently associated with comorbidities, neurological symptoms, and poor functionality [13]. In a second study including COVID-19 patients from the first three waves of the pandemic, a high prevalence of OD and MN was confirmed and a change in the nutritional management of patients from the first to the second wave, with the early application of oral nutritional supplements (ONS), significantly improved clinical outcomes of COVID-19 patients [14].

These results highlight the importance of assessing the effect of FR, OD and MN in COVID-19 patients. Although some studies assessing FR in patients with COVID-19 have emerged [15,16], few of them target a population older than 70 years. The aim of this study was to investigate the role of FR, OD and MN on mortality in hospitalized older patients with COVID-19 during hospitalization and after discharge and to identify preventable or treatable factors in future outbreaks to reduce the high COVID-19 mortality among older patients.

## MATERIAL AND METHODS

### Study design and population

Prospective observational study with COVID-19 patients consecutively admitted to two hospitals of the Consorci Sanitari del Maresme (CSdM), Mataró, Spain: Hospital de Mataró (Acute Care) and Hospital de Sant Jaume i Santa Magdalena (Intermediate Care) during the acute phase of the disease. We assessed patients from April 2020 to January 2021.

A multidisciplinary team comprising nurses, physicians, speech language pathologists (SLPs) and dietitians collected all the information telematically with the patient, their family or with help of the medical staff to minimize the risk of cross-infection according to COVID-19 protocols [17]. Data on patient medical records were collected and patients were telematically followed in the first and third month after discharge. FR and COVID-19 severity were assessed retrospectively.

Inclusion criteria were SARS-CoV-2-infected patients (RT-PCR with GeneXpert Dx [Cepheid, Sunnyvale, CA, USA]) over 70 years of age, admitted to hospital for more than 48h. Patients who were admitted directly to the ICU from the emergency department and died were not included in the study.

The study protocol was approved by the Institutional Review Board (IRB) of CSdM (CEIm 15/21) and was conducted according to the principles and rules laid down in the Declaration of Helsinki and its amendments. Exemption of the informed consent form was granted by the IRB and followed the Guidance on the Management of Clinical Trials during the COVID-19 pandemic (European Commission, version 4; 4 February 2021).

### Study variables

We collected data from patients on admission, during hospitalization, on discharge and 1 and 3 months after discharge.

*Demographic and clinical data:* we registered age, gender, residence and destination on discharge, previous functionality and its evolution (Barthel Index) [18], comorbidities (Charlson Index) [19], COVID-19 severity [20], and hospitalization days.

*Frailty (FR):* we retrospectively collected the FRAIL-VIG index [21,22] using electronic medical records. It is a continuous scale with cut points that differentiate non-frail from frail patients and includes 3 FR levels: mild (FR: 0.2-0.35); moderate (FR: 0.36-0.50) and severe (FR >0.50) [23].

*COVID-19 severity:* we used the COVID-19 illness severity classification of the WHO [20,24]. It categorizes severity of COVID-19 into four levels: mild, moderate, severe, and critical (including acute respiratory distress syndrome (ARDS), septic shock, sepsis and acute thrombosis) [20,24].

*The National Early Warning Score (NEWS-2):* it measures patient deterioration in a range of clinical conditions and settings, and it was measured retrospectively through electronic medical records. It is a predictor of in-hospital mortality and poor outcomes [25] also in COVID-19 patients [26].

*Swallowing evaluation:* we used the Volume-Viscosity Swallowing Test (V-VST) [27], a validated clinical assessment tool to explore safety and efficacy of swallow, simplified for COVID-19 patients with only one volume (10 mL) and 3 viscosities (liquid, 250 mPa·s and 800 mPa·s; prepared with 0 g, 2 g and 5.5 g of Nutilis Clear thickener [Nutricia N.V., Zoetermeer, The Netherlands], respectively, in 100mL water). It was performed by COVID-19 ward nurses with telematic assistance from SLPs [13].

*Nutritional evaluation:* we used the Global Leadership Initiative on Malnutrition (GLIM) Criteria [28,29]. We also registered oral nutritional supplement (ONS) prescription. On admission and discharge we registered blood analytical parameters (albumin, total proteins, cholesterol, ferritin, total lymphocytes and C-reactive protein (CRP). The Reference Laboratory of Catalonia reference intervals were used for comparative purposes [30].

*Mortality:* we collected intra-hospital (IH), 1- and 3-month mortality of study patients. We assessed the effect on mortality of: 1) FR categorized with the FRAIL-VIG index; 2) the presence or absence of OD and/or FR; and 3) the presence or absence of malnutrition (GLIM) and/or FR.

### Statistical and data analysis

Qualitative data were presented as relative and absolute frequencies and continuous as mean and standard deviation (SD). Associations with FR were assessed by the Chi-square test for qualitative data and by the Kruskal-Wallis test for multiple comparisons in continuous data. To assess normality, we used the Kolmogorov-Smirnov test. Multivariate models were made with factors significantly associated and clinically relevant to the different outcomes. We used the Stepwise method to assess the independent factors. For survival analysis, we used the Kaplan Meier method and Log-Rank test to compare curves. Statistical significance was set at p<0.05. Statistical analysis was performed with GraphPad Prism 6 (San Diego, CA, USA) and SPSS v.25.0 (SPSS Inc., Chicago, IL, USA).

**Table 1:** **Demographic and clinical characteristics of the study population according to frailty status**. Bold values indicate p<0.05.

	Total Population	Non-frail	Mild frailty	Moderate frailty	Severe frailty	p-value
**Age (years), ± SD**	82.5±7.6	77.7±6.4	82.9±7.5	86.8±5.8	86.3±6.7	<0.001
**Gender (% women), n/N**	58.9 (152/258)	44.2 (38/86)	70.3 (52/74)	62.9 (44/70)	64.3 (18/28)	0.006
Patient origin, %						
**Community, n/N**	66.3 (171/258)	95.3 (82/86)	77.0 (57/74)	30.0 (21/70)	39.3 (11/28)	<0.001
**Nursing home, n/N**	32.6 (84/258)	4.7 (4/86)	21.6 (16/74)	67.1 (47/70)	60.7 (17/28)	
**Intermediate care hospital, n/N**	1.2 (3/258)	0.0 (0/86)	1.4 (1/74)	2.9 (2/70)	0.0 (0/28)	
**Mean Charlson score, ± SD**	5.3±1.7	4.2±1.1	5.5±1.6	6.0±1.7	6.3±1.5	<0.001
**Mean Barthel Index (pre-admission), ± SD**	64.9±18.0	96.5±8.5	81.6±16.5	57.2±25.3	24.3±21.5	<0.001
**Mean Barthel Index (admission), ± SD**	60.3±34.5	87.4±19.9	61.0±29.4	40.3±27.9	15.4±18.3	<0.001
**Slight dependence (91 - 100), n/N**	28.7 (69/240)	64.3 (54/84)	20.3 (14/69)	1.6 (1/61)	0.0 (0/26)	<0.001
**Moderate dependence (61 - 90), n/N**	21.3 (51/240)	20.2 (17/84)	29.0 (20/69)	23.0 (14/61)	0.0 (0/26)	
**Severe dependence (21 - 60), n/N**	31.7 (76/240)	14.3 (12/84)	40.6 (28/69)	45.9 (28/61)	30.8 (8/26)	
**Total dependence (0 - 20), n/N**	18.3 (44/240)	1.2 (1/84)	10.1 (7/69)	29.5 (18/61)	69.2 (18/26)	
**COVID-19 severity, %**						
**Mild, n/N**	15.1 (39/258)	8.1 (7/86)	12.2 (9/74)	24.3 (17/70)	21.4 (6/28)	<0.001
**Moderate, n/N**	12.8 (33/258)	12.8 (11/86)	16.2 (12/74)	12.9 (9/70)	3.6 (1/28)	
**Severe, n/N**	25.5 (65/258)	11.6 (10/86)	23.0 (17/74)	32.9 (23/70)	53.6 (15/28)	
**Critical, n/N**	46.9 (121/258)	67.4 (58/86)	48.6 (36/74)	30.0 (21/70)	21.4 (6/28)	
**Mean NEWS-2, ± SD**	4.4±6.2	5.2±10.1	4.2±2.4	3.9±2.5	4.1±2.9	0.866
**Length of hospital stay (days), ± SD**	14.0±11.2	16.8±14.2	14.2±9.7	12.2±8.8	10.1±1.7	0.022
Patient destination, %						
**Community, n/N**	59.3 (118/199)	91.1 (72/79)	56.7 (34/60)	22.0 (11/50)	10.0 (1/10)	<0.001
**Nursing home, n/N**	30.7 (61/199)	3.8 (3/79)	28.3 (17/60)	66.0 (33/50)	80.0 (8/10)	
**Intermediate care hospital, n/N**	10.1 (20/199)	5.1 (4/79)	15.0 (9/60)	12.0 (6/50)	10.0 (1/10)	

NEWS-2: the National Early Warning Score. n/N: n represents the number of individuals in the group and N, the total number of individuals.

## RESULTS

### Demographic and clinical characteristics of the study population

We included 258 patients in the study with a mean age of 82.5±7.6 years; 58.9% (n=152) women. Table 1 shows the baseline characteristics of our study population: most came from the community, followed by nursing homes and intermediate-care centers (ICC); hospital stay was 14.0±11.2 days; on discharge, admission to ICC increased from baseline; patients prior to hospital admission and during hospitalization had moderate to severe dependence (Barthel Index 64.9±18.0 and 60.3±34.5, respectively), and also presented a high number of comorbidities (Charlson Index 5.3±1.7).

### Frailty

Up to 66.7% of COVID-19 study patients (n=172) were frail according to the Frail-VIG index. Distribution across FR categories was: 33.3% non-frail (NF), 28.7% mild frail (MF), 27.1% moderate frail (ModF) and 10.9% severe frail (SF). Table 1 shows the baseline clinical characteristics of the study population across FR categories. Regarding the age of the participants, it increased from NF (77.7±6.4 years) to ModF and SF patients (86.8±5.8 and 86.3±6.7 years, respectively) (p<0.001). SF and ModF patients were admitted more frequently from nursing homes than less frail patients that came predominantly from the community (p<0.001). Functionality (Barthel Index) prior to and on admission significantly decreased across FR categories from NF to SF patients (p<0.001), while comorbidities increased (p<0.001). COVID-19 severity (from mild to severe) significantly increased following a gradient from NF to SF patients (p<0.001). However, patients classified with critical COVID-19 severity showed a different pattern, following a decreasing gradient from patients in the NF group to those in the SF group. No differences between FR categories were found in the NEWS-2 scale. Length of hospital stay significantly decreased with FR (p=0.022) and those patients with higher FR severity were more frequently discharged to nursing homes (p<0.001).

### Swallowing function

OD had been previously diagnosed in 19.0% of the study population and its prevalence significantly increased across FR stages (from 3.5% in NF to 42.9% in SF) (p<0.001). Prevalence of OD on admission was 64.4% and also significantly increased across FR stages (NF: 29.80%; MF: 71.6%; ModF: 90.0%; and SF: 96.2%; p<0.0001). SF patients had the highest prevalence of impaired efficacy and safety of swallow (96.2%) (p<0.001) and thus had the greatest need for fluid adaptation with higher viscosities (p<0.001). The same was true for mastication impairments (p<0.001) and the need for texture modified diets (p<0.001) (Table 2).

**Table 2:** **Swallowing function of study population according to the volume viscosity swallowing test (V-VST) categorized by frailty status**. Bold values indicate p<0.05.

	Total Population	Non-frail	Mild frailty	Moderate frailty	Severe frailty	p-value
**Previous OD (%), n/N**	19.0 (49/258)	3.5 (3/86)	17.6 (13/74)	30.0 (21/70)	42.9 (12/28)	<0.001
**OD admission (%), n/N**	65.4 (166/254)	29.8 (25/84)	71.6 (45/74)	90.0 (63/70)	96.2 (25/26)	<0.001
**Impaired efficacy of swallow (%), n/N**	64.0 (162/253)	29.8 (25/84)	68.5 (50/73)	88.6 (62/70)	96.2 (25/26)	<0.001
**Impaired safety of swallow (%), n/N**	53.8 (136/253)	13.1 (11/84)	53.4 (39/73)	87.1 (61/70)	96.2 (25/26)	<0.001
**Fluid adaptation requirement (%), n/N**	49.6 (127/256)	10.5 (9/86)	45.9 (34/74)	84.3 (59/70)	96.2 (25/26)	<0.001
**<50 mPa·s (%), n/N**	50.4 (129/256)	89.5 (77/86)	54.1 (40/74)	15.7 (11/70)	3.8 (1/26)	<0.001
**250 mPa·s (%), n/N**	42.2 (108/256)	9.3 (8/86)	40.5 (30/74)	71.4 (50/70)	76.9 (20/26)	
**800 mPa·s (%), n/N**	7.4 (19/256)	1.2 (1/86)	5.4 (4/74)	12.9 (9/70)	19.2 (5/26)	
**Impaired mastication) (%), n/N**	69.5 (178/256)	40.7 (35/86)	73.0 (54/74)	91.4 (64/70)	96.2 (25/26)	<0.001
**Normal diet (%), n/N**	23.6 (60/254)	51.2 (43/84)	17.6 (13/74)	4.3 (3/70)	3.8 (1/26)	<0.001
**Fork mashable diet (%), n/N**	32.7 (83/254)	38.1 (32/84)	41.9 (31/74)	27.1 (19/70)	3.8 (1/26)	
**Puree diet(%), n/N**	43.7 (111/254)	10.7 (9/84)	40.5 (30/74)	68.6 (48/70)	92.3 (24/26)	

OD: oropharyngeal dysphagia. n/N: n represents the number of individuals in the group and N, the total number of individuals.

### Nutritional status

MN was prevalent in the study population (50.6%) with a relevant weight loss during hospitalization of 5.0±4.8Kg, especially among SF, but no significant differences were observed between FR categories nor for the majority of nutritional parameters studied (Table 3). In addition, ONS were frequently prescribed (93.3%). The overall rate of nasogastric tube placement was 7.2% and was higher as FR decreased, with significant differences between the other groups. We observed reduced albumin and total protein values, at the lower limit of normal standard values in all patients, without significant differences among FR categories, although SF patients presented lowest analytical values (Table 3).

**Table 3:** Nutritional status of study population classified according to frailty status. Bold values indicate p<0.05.

	Total Population	Non-frail	Mild frailty	Moderate frailty	Severe frailty	p-value
**MN GLIM Criteria, (%), n/N**	50.6 (120/237)	50.6 (42/83)	47.1 (32/68)	52.5 (32/61)	56.0 (14/25)	0.869
**Mean BMI (kg/m^2^), ± SD**	28.8±4.0	28.5±4.3	28.7±5.7	27.9±6.3	30.1±2.9	0.776
**ONS prescription, (%yes), n/N**	93.3 (194/208)	90.5 (67/74)	95.3 (61/64)	92.3 (48/52)	100.0 (18/18)	0.444
**NGT placement, (%yes), n/N**	7.2 (18/249)	13.6 (11/81)	7.0 (5/71)	2.9 (2/69)	0.0 (0/28)	0.029
**Weight loss hospitalization (kg) (mean ± SD)**	5.0±4.8	5.5±4.8	4.5±5.3	4.0±4.2	7.5±5.8	0.254
**> 10 kg, (%), n/N**	12.4 (15/121)	12.9 (8/62)	12.5 (4/32)	8.3 (2/24)	33.3 (1/3)	0.516
**6 - 10 kg, (%), n/N**	19.0 (23/121)	24.2 (15/62)	12.5 (4/32)	12.5 (3/24)	33.3 (1/3)	
**3 - 6 kg, (%), n/N**	25.6 (31/121)	30.6 (19/62)	25.0 (8/32)	16.7 (4/24)	0.0 (0/3)	
**1 - 3 kg, (%), n/N**	24.8 (30/121)	17.7 (11/62)	31.3 (10/32)	33.3 (8/24)	33.3 (1/3)	
**< 1 kg, (%), n/N**	18.2 (22/121)	14.5 (9/62)	18.8 (6/32)	29.2 (7/24)	0.0 (0/3)	
Analytical parameters						
**Albumin (g/dL)**	3.0±0.5	3.2±0.4	3.2±0.6	3.1±0.5	2.6±0.6	0.142
**Total proteins (g/dL)**	6.0±0.6	5.9±0.9	6.1±0.6	6.0±0.4	5.9±0.6	0.939
**Lymphocytes (1x10^3^ µL)**	1.4±1.3	1.3±0.6	1.8±3.1	1.4±0.8	1.2±0.6	0.808
**Cholesterol (mg/dL)**	161.6±49.4	178.2±54.1	157.0±44.7	149.6±49.3	NA	0.330

MN: malnutrition; GLIM: global leadership initiative on malnutrition; BMI: body mass index; ONS: oral nutritional supplements; NGT: nasogastric tube; NA: not available. n/N: n represents the number of individuals in the group and N, the total number of individuals.

### Interaction between frailty, oropharyngeal dysphagia and malnutrition

**Figure 1.**
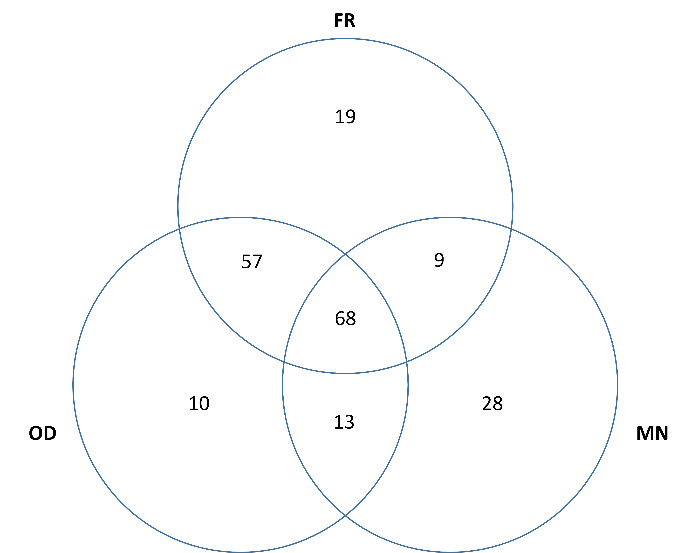
Venn diagram between frailty (FR), oropharyngeal dysphagia (OD) and malnutrition (MN).

Frequencies between FR, OD and MN and its overlapping are shown in Figure 1. It is important to note that a high number of patients had overlapping between the three conditions (68/204 patients), and also those patients with overlapping of two conditions (FR and OD (125/204), OD and MN (81/204), and MN and FR (77/204)). In addition, we found that patients with OD and FR but without MN (57/204) were more frequent than those with OD and MN but without FR (13/204), and those with FR and MN but without OD (9/204)).

### Mortality

**Figure 2.**
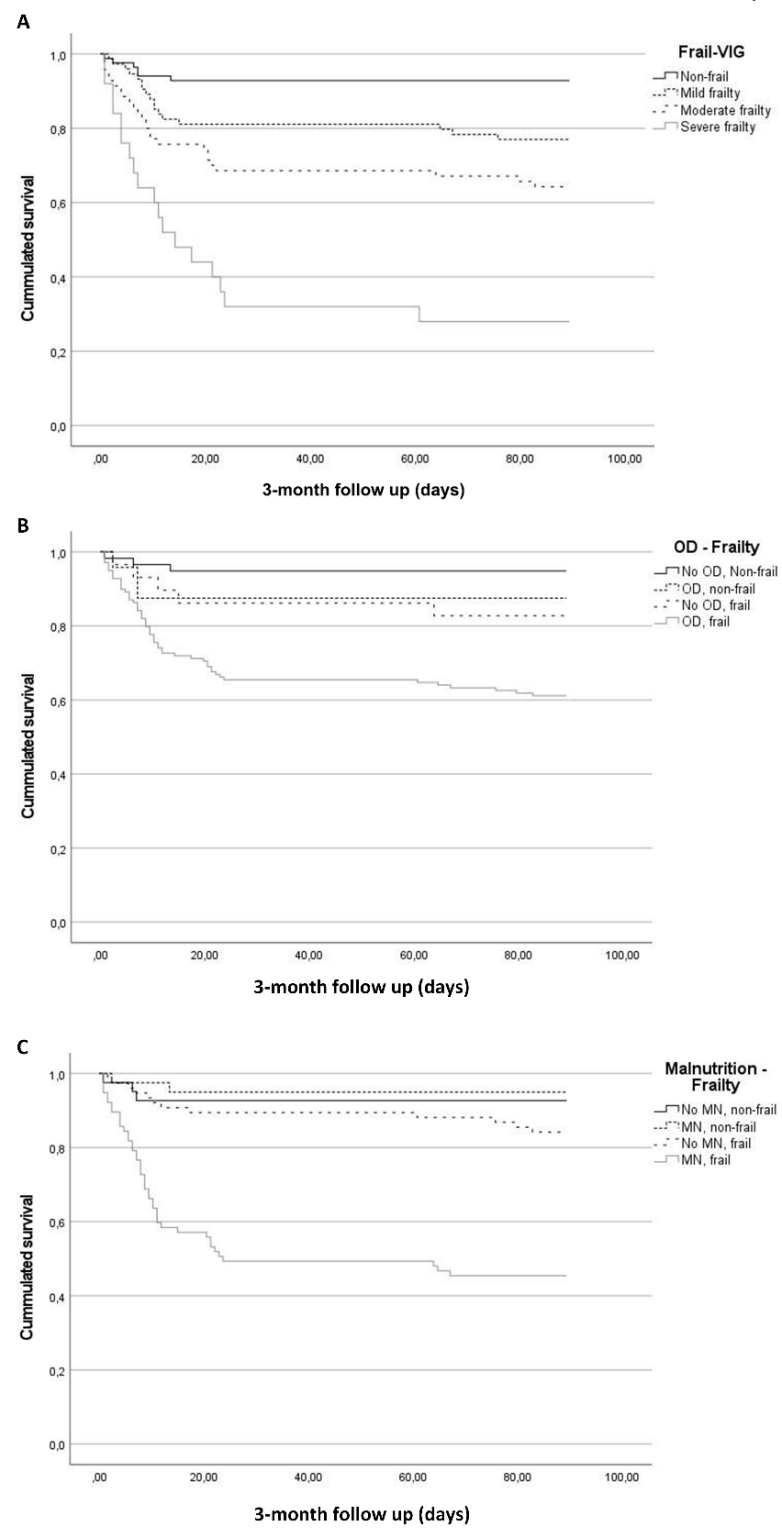
**3-month survival curves**. A) Patients classified according to frailty category, p<0.001; B) Patients classified according to frailty and oropharyngeal dysphagia (OD). p<0.001; and C) patients classified according to frailty and malnutrition (MN), p<0.001.

Mortality over the whole study period was 27.5% (71/258) and increased with FR categories (9.3% NF; 23.0% MF; 35.7% modF; and 75.0% SF; p<0.001) (Fig. 2). Up to 83.1% (59/71) of deceased patients died during hospitalization due to respiratory failure caused by SARS-CoV-2 pneumonia (84.2%), followed by broncho-aspiration (5.3%). No patients died between hospital discharge and 1 month follow up. Of the 12 patients who died after 1-month follow up, 41.6% (5/12) died due to respiratory sequelae secondary to COVID disease, 16.7% (2/12) from sepsis of non-respiratory origin and 8.3% (1/12) from acute heart disease and bronchoaspiration. Cause for death was not recorded in 3 patients.

IH and 3-month mortality increased across FR categories (IH: 8.1% NF, 19.2% MF, 27.1% modF, and 67.9% SF; p<0.001. 3-months: 1.6% NF, 6.4% MF, 14.0% modF, and 50.0% SF; p<0.001, respectively) (Fig. 2).

*Oropharyngeal dysphagia:* Overall mortality increased from non-frail patients without OD to those who had both conditions (6.8% no OD+no FR; 16.0% OD+no FR; 17.2% no OD+FR; and 39.7% OD+FR; p<0.001) (Fig. 2). IH mortality followed a similar pattern (6.8% no OD+no FR; 12.0% OD+no FR; 13.8% no OD+FR; and 32.9% OD+FR; p<0.001). 3-month mortality was higher in patients with OD and FR (0.0% no OD+no FR; 5.9% OD+no FR; 5.9% no OD+FR; and 13.9% OD+FR; p=0.021).

*Malnutrition:* Overall mortality increased from non-frail patients without MN to those who had both conditions (7.3% no MN+no FR; 9.5% MN+no FR; 15.8% no MN+FR; and 55.1% MN+FR; p<0.001) (Fig. 2). IH mortality was similar in patients with or without one of the two conditions but was significantly higher in those who had both (7.3% no MN+no FR; 7.1% MN+no FR; 7.9% no MN+FR; and 50.6% MN+FR; p<0.001). Finally, 3-month mortality was higher in frail patients (0.0% no MN+no FR; 3.1% MN+no FR; 12.7% no MN+FR; and 12.5% MN+FR; p=0.110).

**Figure 3.**
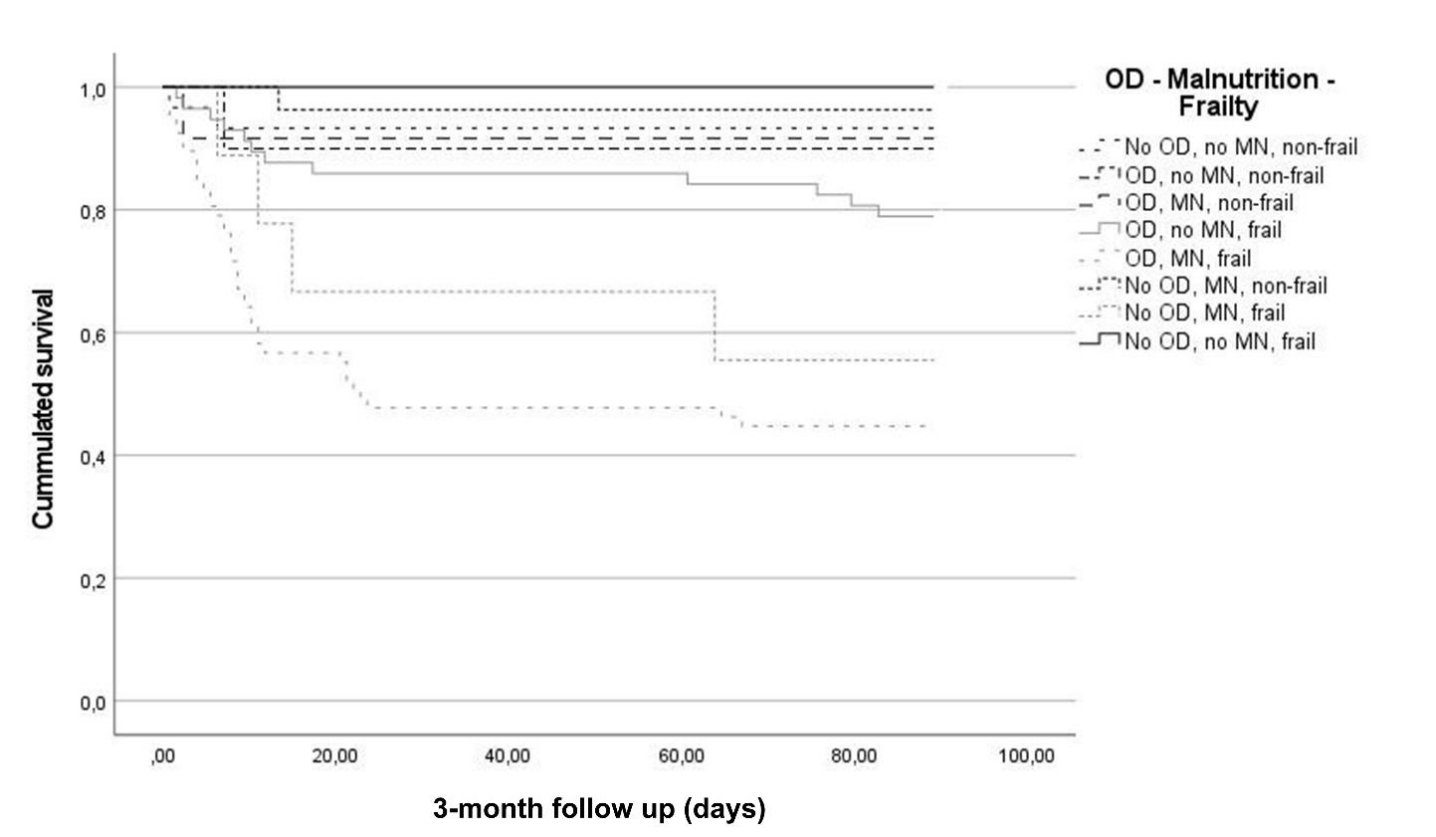
3-month survival curve of patients classified according to frailty, oropharyngeal dysphagia (OD) and malnutrition (MN), p<0.001.

*Frailty, oropharyngeal dysphagia, and malnutrition:* The combination of OD, MN and FR had the worst prognosis among study patients with a 3-month mortality of 55.22%, followed by those without OD and with MN and FR (44.4%) and those without MN and with OD and FR (21.1%) (p<0.001) (Fig. 3).

### Multivariate analysis

A multivariate analysis adjusted by age, sex, functionality (Barthel pre-admission), comorbidities (Charlson score), patient origin, nutritional status (MN with GLIM criteria), dysphagia, FR (FRAIL-VIG), and COVID-19 severity was carried out. It showed that the presence of impaired pre-admission functional capacity (HR=0.983, CI-95%:0.973-0.993; p=0.001) and MN during hospitalization (HR=4.279, CI-95%:1.658-11.049; p=0.003) were independently associated with IH mortality. OD was closely associated with IH mortality but did not reach statistical significance (HR=2.953, CI-95%:0.970-8.989; p=0.057).

## DISCUSSION

The aim of this study was to investigate the role of FR, OD and MN on mortality in hospitalized older patients with COVID-19 during hospital stay and after discharge. We evaluated 258 patients, with a mean age of 82.5 years admitted to a general hospital and found a high prevalence of FR (66.7%), OD (65.4%) and MN (50.6%). Overall IH-mortality was 23.0% and an additional 6.6% during 3-month follow up. The main causes of mortality were respiratory complications associated with COVID-19 and bronchoaspiration. We found that FR, OD and MN were associated with IH and 3-month mortality. Finally, Barthel pre-admission, MN and OD (p=0.057) were independent risk factors for IH mortality.

Overall mortality rate in this study was 27.5% and increased to 75% in SF patients. In a previous study, with a younger cohort of patients from the first wave of the pandemic, we found an overall mortality rate of 16.6% over 6-month follow up [13]. Another study, with younger individuals, found an IH mortality of 10.7%, 13.6% and 19.1% over the first, second and third waves, respectively [14]. Several studies have shown widely divergent mortality rates: 34.0% (IH) in Canada [31], 11.0% in China [2], and 11.4%, 19.8% and 18.0% (IH) in Spain [32-34]. The differences found are due to differences in patient characteristics or patient management during the pandemic. However, when FR is taken into account, mortality increases exponentially, revealing the impact of this condition. Mortality and poor outcomes among COVID-19 older adults has been found to be independently associated with patient age, BMI, male gender, dementia, impaired functionality, chest X-ray consolidation, hypoxemic respiratory failure, and lower oxygen saturation on admission (6). We also found that OD (p=0.057) and MN were independent risk factors for IH mortality. OD is not always assessed in COVID-19 and thus might have not been included in other studies on COVID-19 mortality. We previously found that OD was highly prevalent in the general COVID-19 population and significantly associated with MN, impaired functionality, institutionalization and intra-hospital and 6 months mortality [13,14], showing the relevance of OD assessment and treatment in these patients. Finally, a systematic review recommended strict preventive measures, timely diagnosis, and aggressive therapeutic and nontherapeutic care to improve COVID-19 frail patient outcomes [6].

We included a cohort of older patients (82.5±7.6 years), mainly hospitalized from the community, who had impaired functionality and a high number of comorbidities. The majority of them had severe (25.5%) or critical COVID-19 (46.9%). Our population age is higher than the majority of the published studies as we included only older patients of more than 70 years [13,14]. Overall length of hospital stay (14.0±11.2 days) was lower than a previous study we published on the first wave of the pandemic (16.8±13.0 days) [13] and of Chinese studies (14 days (IQR 10-19)) [35], but higher than that of another of our previous studies with patients from the first, second and third waves (12.7±10.9 days) [14] and to those reported in a systematic review which showed a mean 5 days (IQR 3-9). These disparate results may be explained by differences in admission and discharge criteria between countries, different periods of the pandemic and age and phenotypical characteristics of study participants.

FR was highly prevalent in our study population (66.7%), a strong indicator of impaired health status and associated with OD and mortality. As FR severity increased, patients were older, more frequently institutionalized on discharge, had more comorbidities, impaired functionality and more severe COVD-19 disease. Interestingly, OD had more interaction with FR than MN (Fig. 1), probably due to the similar impairment of MN in all studied patients independently of FR categories, and the association of FR, functional deterioration and age on OD [13,14]. Another study found that frail COVID-19 patients were significantly older than robust individuals and had longer hospital stay [36]. SF patients had the lowest percentage of critical patients and the shortest hospital stay. This could be explained because, due to the excess demand during the pandemic, non-frail patients and those with fewer comorbidities were given priority to ICU admission (for critical patients) as they had better chance of survival (https://semicyuc.org/2021/07/revision-guias-covid-19/). Regarding the shorter hospital stay, mortality among SF patients was higher and they also had higher rates of hospital discharge to post-acute or long-term care centers where they continued their recovery, while most non-frail patients were discharged home after recovery. This was consistent with the results of a multicenter British study [37].

The pathophysiology of OD related to COVID-19 is related to sensory dysfunction caused by several neurological impairments associated with SARS-CoV-2 infection and to the invasion of peripheral nerves by the virus leading to ageusia, anosmia and sensory impairment [4,38]. Respiratory insufficiency, invasive ventilatory procedures, MN, sarcopenia, cachexia and dysfunctionality have also been related to COVID-19-induced OD [3,4]. Prevalence of OD during hospitalization was very high (65.4%; 47.6% newly diagnosed patients). OD prior and during hospital admission was associated with FR and increased with its severity (13.1% NF to 96.2% SF). We had previously found that OD was highly prevalent in hospitalized COVID-19 patients (51.7%, 31.3% and 35.1% in the first, second and third waves of the pandemic, respectively) [13,14]. We now found a higher prevalence due to increased mean age and FR. Other studies reported lower prevalence of OD, 28.9% in older patients, 67.6±17.6 years [39] and 26.9% in post-extubated patients with COVID-19 [40]. These differences could be related, again, to the age of the participants, to other patient characteristics such as functionality and comorbidities, or to the swallowing assessment method used. In our case we used an adapted version of the V-VST [13,14], a clinical assessment with good psychometrics [27,41]. A recent meta-analysis on the prevalence of OD in 2055 COVID-19 patients concluded that its prevalence was 35.0% and that OD assessment among these patients deserves more attention [42].

OD is associated with severe complications such as MN, dehydration, respiratory infections and mortality [43]. In fact, in this study we found a 5.3% and 8.3% of IH and 3-month mortality, respectively, due to bronchoaspirations, that can be directly attributed to OD. We found a similar result in a previous study during the first wave of the pandemic -the most severe- with 4.5%, and 25.0% IH and 3-month mortality due also to bronchoaspirations in COVID-19 patients. Moreover, OD was found to be an independent risk factor (p<0.0001) for mortality in the same institution [14]. A study reported that 96% of COVID-19 patients assessed with fiberoptic endoscopic evaluation of swallow (FEES) after extubation had signs of impaired safety of swallow and recommended evaluating the swallowing function as standard procedure, preferably at an early stage, before initiation of oral intake to avoid complications related to dysphagia [44]. According to these recommendations, mortality could have been avoided by directly treating OD with simple and cost-effective tools and interventions such as the V-VST for clinical diagnosis [41] and adaptation of fluids and solids with thickeners and texture-modified diets, respectively, to minimize aspirations: minimal-massive intervention (MMI) [45]. We previously introduced a multimodal intervention based on texture adaptation of alimentary fluids and solid foods, and the systematic prescription of ONS to all patients admitted with COVID-19 to our hospital from the second wave forward (2 units; each bottle 200mL and 300kcal+15g protein) from admission and use of fortified and texture-modified foods during hospitalization and 6-month follow-up. After the intervention, we found significant improvements in several nutritional parameters versus patients from the first wave [14].

Due to the prevalence of OD in the COVID-19 older population and the high risk of infection for healthcare professionals, identifying patients most at risk of OD is important. To this end, we have recently developed an artificial intelligence tool with good psychometric properties to assess the risk of OD in patients and to prioritize which ones who need clinical evaluation first to start MMI treatment and ensure their hydration and nutritional intake [13, 46].

Prevalence of MN and weight loss (WL) among older COVID-19 patients was high. However, none of the nutritional parameters presented significant differences across FR categories. In contrast, a study from Damanti S et al. [36] reported that FR patients were more malnourished than robust people one month after discharge. This is mainly due to the high impact of COVID-19, independently of its severity, on the nutritional status of patients due to loss of appetite and long hospital stay leading to disuse atrophy associated with muscle loss, high muscle catabolism, hypermetabolism and systemic inflammation [47,48]. Nevertheless, our results suggest worse nutritional status in patients with SF (higher prevalence of MN, total WL and percentage of WL, and decreased nutritional blood parameters) that might have been significant in a bigger study population. Risk of MN, MN and WL in COVID-19 disease has been reported to be high in several studies, indicating the impact of this disease on nutritional status [13,14,49,50]. One surprising result was the placement of nasogstric tube (NGT) which was highest in non-FR patients and decreased across FR categories. This could be related to several factors such as age and functional deterioration (non-FR were younger and more independent) and ICU admission protocol as NGT were placed in ICU during the mechanical ventilation treatment and subsequently used to nourish, and SF patients were less likely to be admitted to ICU.

FR has been found to be a strong predictor of mortality and poor outcomes among COVID-19 patients with a difference between 67.9% vs 8.1%, 50.0% vs 1.6% and 75.0% vs 9.3% in severe vs non-frail patients for IH, 3-months and overall mortality rate, respectively. As reported in this study and others, mortality increased across FR categories, being maximal in those with more severe FR status [37,51,52]. A systematic review and meta-analysis on the relationship between FR and mortality in 3817 COVID-19 patients of similar age to our study population found similar results, showing that increase in clinical FR scale was associated with increase in mortality in a linear manner [52]. Another study found that age and FR were independently associated with adverse outcomes in COVID-19 patients, and patients that survived had more care requirements than non-FR or younger patients [37]. Another study in a cohort of 368 COVID-19 patients found that age was independently associated with 1-year mortality [53]. In our study we have not found independent associations of age with mortality, however, we only included patients above 70 years old and inside this age group, mortality was associated with other factors such as functionality, nutrition and swallowing function.

We have also found that OD and MN increased patients' overall and IH mortality, especially when they were combined with FR. The combination of FR, OD and MN had the worst prognosis, with a 3-month mortality of 55.2%. Finally, we found that pre-admission functionality, MN, and OD (p=0.057) were independent risk factors for IH mortality in COVID-19 patients. We consider OD to be an independent risk factor for IH mortality because we previously found it in a COVID-19 population of 605 patients in the same hospital [14] and the low p-value needed to achieve statistical significance (0.0071). A meta-analysis also found that OD was associated with a high risk of mortality in COVID-19 patients [42]. OD and FR showed some overlapping or collinearity (Fig. 1, supplementary Tables 1 and 2), however our multivariate analysis (step wise method) excluded FR in the final model. The fact that these risk factors are easy to treat, especially OD and MN, reinforces our call for early screening, assessment and treatment of these conditions with simple and effective measures [45].

The study presents some limitations: 1) there is no gold standard for FR assessment, partly due to the lack of consensus over its definition, and no specific tool has proven to be the best method for COVID-19 patients. However, we used the Frail-VIG index, a tool validated in Catalonia, the same region as our hospital, in people of similar age to our patients [23]. This index is a reliable, reproducible and feasible instrument with a positive correlation with the Clinical Frailty Scale (CFS) [54]. It was a good prognosis estimator in our sample. 2) Previous diseases of study participants were not registered individually and thus we were not able to assess the effect of these comorbidities on frailty, swallowing and nutritional status and mortality. Instead, we registered the Charlson comorbidity index, but we found it was not independently associated with intrahospital mortality. 3) The telematic evaluation and follow up of patients precluded a more comprehensive assessment and may have affected the prevalence described in the study, but the risk of infection of study investigators justified the use of these tools. The use of an instrumental assessment method such as videofluoroscopy (VFS) or fiberoptic endoscopic evaluation of swallow (FEES) would have given us more information on signs of impaired efficacy and safety of swallow of study participants as well as quantitative information of the oropharyngeal swallow response (OSR). However, according to an European Guideline of the European Society for Swallowing disorders, instrumental assessment was not recommended due to high risk of infection to healthcare professionals and was only recommended in particular situations: only if a potential life-threatening underlying disease was suspected, clinical assessment did not provide enough diagnostic information for effective treatment to be prescribed to the patients and the clinical situation does not allow the clinical decision to be postponed [55]. 4) FR and COVID-19 severity were assessed retrospectively from the electronic medical history of patients due to the high hospital care load of clinicians and researchers during the pandemic.

### Conclusions

FR, OD and MN are highly prevalent conditions in older COVID-19 patients. FR is associated with impaired health status and functionality, and it is a strong predictor of mortality and poor outcomes in patients with COVID-19; however, it is rarely measured. OD and MN are also related to poor outcomes in COVID-19 patients [13,14]. Clinicians would benefit from a standardized assessment of FR, OD and MN in hospitalized patients and their inclusion in clinical guidelines to make comprehensive clinical decisions regarding therapeutic approaches and early rehabilitation strategies for these conditions. We recommend early, proactive and aggressive multimodal interventions to reduce the high lethality of COVID-19 among older patients.

## Supplementary Materials

The Supplementary data can be found online at: www.aginganddisease.org/EN/10.14336/AD.2023.0425-2.


