# Supplementary material for: The Impact of Frailty, Oropharyngeal Dysphagia and Malnutrition on Mortality in Older Patients Hospitalized for Covid-19
Source: Aging Dis. 2024 Apr 1;15(2):927–38. doi: 10.14336/AD.2023.0425-2 (PMC10917529; doi:10.14336/AD.2023.0425-2)
Supplement: Supplementary file 1 [file AD-15-2-927-s.pdf]

SUPPLEMENTARY DATA

The Impact of Frailty, Oropharyngeal Dysphagia and Malnutrition on Mortality in Older Patients Hospitalized for Covid-19

Alberto Martín-Martínez, Paula Viñas, Irene Carrillo, Josep Martos, Pere Clavé, Omar Ortega

© 2023. Martín-Martínez A et al. Published online at http://www.aginganddisease.org/EN/10.14336/AD.2023.0425-2

SUPPLEMENTARY DATA

Supplementary Table 1. Multivariate analysis for intra-hospital mortality showing HR (CI-95%) for oropharyngeal dysphagia (OD), malnutrition (MN) and frailty (FR). In bold the values that are affected when a combined analysis (enter method and stepwise method) is performed (OD and FR), showing some collinearity. Note that in the step wise method, the one used in the study, FR is taken out from the analysis.

	INDIVIDUAL EFFECT OF VARIABLES	ENTER METHOD (Single step)	STEP WISE METHOD
OD	**3.3 (1.4 – 7.8)**	**2.1 (0.6 – 7.2)**	2.9 (0.97 – 9.0)
MN	4.6 (1.8 – 11.9)	4.4 (1.7 – 11.7)	4.3 (1.66 – 11.0)
FR	**4.6 (1.8 -11.7)**	**2.8 (0.7 – 11.8)**	-

Supplementary Table 2. Chi-square tests between paired variables (oropharyngeal dysphagia (OD), malnutrition (MN) and frailty (FR)).

	OD	no OD	p-value
FR %	84.9	33.0	<0.001

	MN	no MN	p-value
FR %	65.0	65.0	0.994

	MN	no MN	p-value
OD %	66.9	57.8	0.144
